# Phosphorylated tau 217 in young adults with Down syndrome and Down syndrome regression disorder

**DOI:** 10.1038/s43856-026-01921-y

**Published:** 2026-09-21

**Authors:** Jonathan D. Santoro, Sara Abdel-Latif, Abhik K. Banerjee, Hannah Mager, Mackenzie Silverman, Lilia Kazerooni, Samuel T. Otey, Mariam M. Yousuf, Maeve C. Lucas, Zinayida Schlachetzki, Robert A. Rissman, Michael S. Rafii

**Affiliations:** 1 Division of Neurology, Department of Pediatrics, Children’s Hospital Los Angeles, Los Angeles, CA USA; 2 Department of Neurology, Keck School of Medicine of the University of Southern California, Los Angeles, CA USA; 3 Alzheimer’s Therapeutic Research Institute, University of Southern California, San Diego, CA USA; 4 National Institutes of Health, Bethesda, MD USA

**Keywords:** Neurodevelopmental disorders, Diagnostic markers

## Abstract

**Background:**

Down syndrome regression disorder (DSRD) causes a rapid loss of cognitive, behavioral, and daily living skills, usually in adolescents and young adults with Down syndrome. Because people with Down syndrome have a high lifetime risk of Alzheimer’s disease, we examined whether DSRD is associated with an Alzheimer-related pattern of tau pathology.

**Methods:**

In this prospective, single-center study, we measured plasma phosphorylated tau-217 (p-tau217), a blood marker of Alzheimer pathology, in 132 participants: 48 with DSRD, 39 with Down syndrome without regression (DSwR), and 45 neurodevelopmental controls. We compared p-tau217 concentrations across groups and examined relationships with demographic factors, medical conditions, treatment status, and diagnostic test abnormalities.

**Results:**

Median p-tau217 concentrations were similar in the DSRD (0.082 pg/mL), DSwR (0.084 pg/mL), and neurodevelopmental control (0.080 pg/mL) groups (overall comparison, *p*  =  0.57). Pairwise comparisons and models accounting for demographic and clinical factors also showed no statistically significant group differences. Among participants with DSRD, p-tau217 was not associated with treatment status or abnormalities on brain-wave testing, neuroimaging, or cerebrospinal fluid analysis. No clinical or demographic factor remained associated with p-tau217 after accounting for the number of analyses performed.

**Conclusions:**

These findings provide no evidence of a group-level Alzheimer-related p-tau217 elevation in young people with DSRD and support DSRD as a condition distinct from Alzheimer’s disease. However, a single blood measurement cannot exclude Alzheimer pathology in an individual. Longitudinal studies using several complementary biomarkers are needed.

## Plain language summary

People with Down syndrome have a high lifetime risk of Alzheimer’s disease, a neurodegenerative disorder that affects memory, thinking, and behavior. Down syndrome regression disorder (DSRD) causes sudden changes in thinking and behavior, usually in adolescents and young adults. We asked whether DSRD involves the same brain changes seen in Alzheimer’s disease. We measured p-tau217, a blood marker linked to Alzheimer’s disease, in 132 people with DSRD, Down syndrome without regression, or other developmental conditions. Levels were similar in all three groups and were not related to DSRD treatment or common medical conditions. These findings suggest that Alzheimer’s-related changes are unlikely to be the main cause of DSRD and support research that examines other causes besides those related to Alzheimer’s disease, including immune-related changes. Future studies should measure this marker repeatedly over time in larger groups.

## Abstract (web summary)

Santoro et al. compare plasma p-tau217 concentrations in individuals with Down syndrome regression disorder, Down syndrome without regression, and typical neurodevelopment as controls. P-tau217 levels do not differ across groups, suggesting against Alzheimer-related tau pathology as a primary driver of DSRD.

## Introduction

Down Syndrome Regression Disorder (DSRD) is an increasingly recognized clinical syndrome marked by the rapid loss of previously acquired cognitive, behavioral, and adaptive skills in adolescents and young adults with Down syndrome (DS)^1–4^. The abrupt onset and broad neuropsychiatric features of DSRD often prompt clinicians to consider a wide differential diagnosis^5,6^, including neurodegenerative conditions such as Alzheimer’s disease (AD). Individuals with DS have an increased risk of AD over the lifespan^7,8^, and given certain clinical similarities, some have speculated that DSRD may be on the AD spectrum.

Despite this association, there is currently no evidence to support AD as the underlying cause of DSRD. Notably, DSRD differs from typical AD in its subacute presentation, symptom profile, and potential for partial reversibility^9–11^. Clinically, anchoring regression to AD risk may lead to misclassification, delayed evaluation for potentially treatable etiologies, and inappropriate counseling regarding prognosis.

Blood-based biomarkers have begun to transform AD diagnostics by enabling biologically informed risk stratification and differential diagnosis without requiring cerebrospinal fluid or advanced neuroimaging in all patients^12^. In particular, phosphorylated tau species, including p-tau217, have shown strong performance for identifying AD-related tau pathology across multiple cohorts and are increasingly used as markers of AD biology^13,14^. Although there is no standard for the diagnosis of AD based on p-tau217 levels, a cutoff of 0.4 pg/mL has been proposed as an intermediate risk zone that should prompt additional investigations^14^. Whether DSRD is accompanied by an AD-like biomarker signature remains uncertain, and clarifying this question is clinically relevant for diagnostic algorithms, family counseling, and mechanistic framing of DSRD as neurodegenerative versus neuroimmune/neuropsychiatric.

In this study, we compared plasma p-tau217 concentrations among individuals with DSRD, individuals with DS without regression (DSwR), and neurodevelopmental (NDD) controls. We further evaluated whether common comorbidities, clinical features and medical history were associated with p-tau217 levels and whether adjustment for these factors meaningfully altered group comparisons. We hypothesized that DSRD would not demonstrate a distinct p-tau217 elevation consistent with an AD phenotype, supporting the view that DSRD is a biologically distinct regression syndrome rather than AD.

Here, median plasma p-tau217 concentrations are similar in DSRD (0.082 pg/mL), DSwR (0.084 pg/mL), and NDD controls (0.080 pg/mL), with no significant overall difference (*p* = 0.57) and no diagnostic group effect after covariate adjustment. Among participants with DSRD, p-tau217 is also not associated with treatment status or neurodiagnostic abnormalities, and no evaluated clinical or demographic factor remains significant after correction for multiple testing. Together, these findings do not support a group-level AD-related p-tau217 elevation in DSRD.

## Methods

### Human ethics and consent to participate

This study was approved by the Children’s Hospital Los Angeles Institutional Review Board (IRB Number: 23-00119) in accordance with the Declaration of Helsinki. Participants were enrolled prospectively with assent (if <18 years of age) and consent (if >18 years of age) obtained when possible. In circumstances where individuals did not have the capacity to assent, consent, or both, a parent, guardian, or legally authorized representative (LAR) was consented in lieu of the participant. In circumstances where individuals could not consent or assent, an independent clinical research monitor reviewed enrollment criteria and interviewed the consenting representative to ensure that no coercion towards participation and fully informed decision-making were present prior to enrollment.

### Patient populations

Patients were recruited from a single center in multiple outpatient clinics. Individuals were identified through both re-contacting from an established research database and ICD-10 code screening. There were three unique patient cohorts in this study.

1. DSRD: Individuals with DSRD of age <26 years were eligible to participate in this study if they had a confirmed diagnosis of trisomy of chromosome 21 by karyotype and they met either possible or probable international consensus criteria for DSRD and a full neurodiagnostic workup had been performed (electroencephalogram, MRI, and lumbar puncture)^5^. ICD-10 codes used for screening for this cohort were: Q90.0, Q90.2, and/or Q90.9.
2. DSwR: Individuals with DSwR of age <26 years were eligible to participate in this study if they had a confirmed diagnosis of trisomy of chromosome 21 by karyotype. ICD-10 codes used for screening for this cohort were: Q90.0, Q90.2, and/or Q90.9.
3. NDD Controls: Individuals with any NDD condition of age <26 years were eligible to participate in this study. Individuals in this cohort had to have focused-exome (NDD or epilepsy-focused gene panel) or whole exome genetic testing in the past five years and could have any non-monogenetic cause of ID and/or autism spectrum disorder (ASD). ICD-10 codes used for screening for this cohort were: F70, F71, F72, F73, F78, F79, F84, F84.1, F84.5, and F84.9.

Exclusion criteria were uniform between all groups and included prior neurosurgical intervention (e.g., shunt placement), prior or active receipt of radiotherapy or chemotherapy, or active cardiac, pulmonary, or renal disease. In addition, individuals with epilepsy in any group were excluded if they had a seizure within one month of the blood draw. Individuals in the NDD control cohort were excluded if they had a monogenetic cause of ID or ASD (e.g., *SHANK3* gene mutation-associated ASD, *MECP2*-associated Rett syndrome, etc.) Finally, individuals with missing data were excluded from the study.

### Assessment of degree of intellectual disability

All groups had an assessment of baseline degree of ID per the Diagnostic and Statistical Manual of Mental Disorders (DSM-5) criteria. For the DSRD cohort, the pre-regression level of ID was utilized.

### Definition of neurodiagnostic abnormalities and treatment in the DSRD cohort

Neurodiagnostic abnormalities were determined based on previously published studies. Reference to definitions is located in Supplementary Table 1. Treatment response was also assessed in the DSRD cohort. Treatment was defined as clinical management with psychotropic medications (antidepressant and/or antipsychotic therapies) or immunotherapy (steroids and/or intravenous immunoglobulin).

### Sample processing

Samples were obtained in addition to routine phlebotomy procedures and not as an isolated lab draw. Blood was collected in EDTA tubes and then centrifuged at 700 × *g* for 15 min. Plasma was then aspirated from the samples and centrifuged a second time at 2200 × *g* for 15 min. Samples were stored at −80 °C.

### p-tau217 processing

Plasma p-tau217 was measured using the fully automated Fujirebio Lumipulse® G1200 instrument and Fujirebio’s commercially available p-tau217 kits. A calibration curve was established to determine sample concentrations by measuring calibrators in duplicate. Quality controls were also measured at the beginning of each analytic session. Prior to analysis, plasma samples were thawed for 30 min at room temperature, vortexed for 10 s, and centrifuged at 2000 × *g* for 5 min. Samples were then loaded into cuvettes for analysis on the instrument.

### Statistical analysis

Participants were categorized into three prespecified cohorts (DSRD, DSwR, and NDD control). The primary outcome was plasma p-tau217 concentration (pg/mL). Analyses were organized hierarchically as primary, sensitivity, adjusted, and exploratory analyses. The primary analysis was the unadjusted comparison of plasma p-tau217 concentrations across the three diagnostic cohorts. Sensitivity analyses evaluated the robustness of this comparison using parametric approaches. Adjusted analyses estimated diagnostic group effects after accounting for prespecified demographic and clinical covariates. Analyses of treatment status, neurodiagnostic abnormalities, individual clinical comorbidities, and elevated p-tau217 thresholds were considered exploratory and hypothesis-generating.

Continuous demographic and clinical variables were summarized descriptively as mean (SD) to facilitate comparison across cohorts, while categorical variables were summarized as *n* (%). Distributional assumptions were assessed for continuous analytic variables using visual inspection of histograms and Q-Q plots, supplemented by the Shapiro–Wilk test when appropriate. As plasma p-tau217 demonstrated right-skewness, p-tau217 values were summarized using median and interquartile range (IQR) and analyzed primarily using nonparametric methods.

For unadjusted between-group comparisons of p-tau217, the primary inferential test was the Kruskal–Wallis test given the right-skewed distribution typical of biomarker outcomes. Parametric tests, including one-way ANOVA and Welch ANOVA, were performed only as sensitivity analyses to assess the robustness of the primary nonparametric findings and were not interpreted as assuming normality of the raw p-tau217 distribution. Pairwise group comparisons, when performed, used Mann–Whitney U tests with Holm correction for multiple comparisons. As sensitivity analyses, one-way analysis of variance (ANOVA) and Welch ANOVA were also computed. Associations between p-tau217 and additional demographic/clinical covariates were assessed using Spearman rank correlation for continuous predictors (e.g., age at collection, BMI) and Mann–Whitney U tests (2 levels) or Kruskal–Wallis tests (≥3 levels) for categorical predictors. To address multiplicity across exploratory covariate-screening analyses, false discovery rate was controlled using the Benjamini–Hochberg procedure. Exploratory analyses not adjusted for multiple testing are explicitly identified as such and were interpreted descriptively, with emphasis on effect sizes, confidence intervals, and consistency across analyses rather than isolated nominal *p* values.

Adjusted group effects were estimated using multivariable linear regression with log-transformed p-tau217 as the dependent variable to improve model fit and permit interpretation on a multiplicative scale. The primary adjusted model was intentionally parsimonious and included covariates selected a priori based on biological relevance to AD biomarker interpretation, blood-based biomarker physiology, or plausible tau-related neuronal injury: age at collection, sex, APOE ε4 carrier status, BMI, and epilepsy/infantile spasms. Age and APOE ε4 carrier status were included because of their established relevance to AD risk and biomarker trajectories; BMI was included because systemic physiology and blood-based biomarker concentrations may vary with body habitus; sex was included as a standard demographic covariate; and epilepsy/infantile spasms was included because seizure-related neuronal injury could plausibly influence tau-related biomarkers.

A broader clinical model additionally including race, ethnicity, autism, cardiac history, sleep apnea, cancer history, immunologic disorder, thyroid disease, psychiatric disease, diabetes, hypertension, degree of ID, and treatment status among DSRD participants was performed as an exploratory sensitivity analysis. This model was intended to assess whether the diagnostic group effect was robust to medical and neurodevelopmental complexity rather than to identify independent predictors among all included covariates. Given the cohort size and number of parameters, individual covariate estimates from this broader model were interpreted cautiously. Robust (HC3) standard errors were used to mitigate heteroskedasticity. Adjusted effects are presented as percentage differences in geometric mean p-tau217 (100 × [exp(β) − 1]) with 95% CIs derived from robust standard errors. Two-sided *p* values < 0.05 were considered statistically significant.

To evaluate whether demographic or clinical characteristics were associated with abnormal plasma p-tau217 levels, we conducted a series of univariable logistic regression analyses. Plasma p-tau217 concentrations were right-skewed; therefore, to create a clinically interpretable binary outcome, p-tau217 was dichotomized to define elevated versus low levels. An exploratory post-hoc cutoff point of 0.125 pg/mL derived from prior work in AD-related clinical syndromes, was used solely to create an above-threshold binary outcome for exploratory logistic analyses^15^. DS–specific plasma p-tau217 thresholds have been proposed and appear higher than euploid thresholds^13^, but they remain assay-specific and are not yet standardized as universal clinical diagnostic cutoffs. Current evidence supports p-tau217 as a strong screening/enrichment and adjunctive diagnostic biomarker for AD pathology in DS, rather than a stand-alone diagnostic test. This percentile-based threshold ensured adequate case representation while avoiding distributional assumptions inappropriate for this biomarker. Odds ratios (ORs) and 95% Wald confidence intervals were calculated for each predictor. Because these univariable logistic regression analyses were exploratory and intended to identify potential clinical correlates of elevated p-tau217 rather than to test a single prespecified hypothesis, results were interpreted descriptively with attention to effect sizes and confidence intervals. To reduce the risk of overinterpreting nominal associations, *p* values from these models were additionally evaluated using the Benjamini–Hochberg false discovery rate procedure as a sensitivity analysis. Continuous variables (age, BMI) were standardized to one standard deviation to allow comparability of effect sizes across variables measured on different scales. Variables with insufficient cell counts (e.g., hypertension) were excluded from modeling due to unstable parameter estimation. For evaluation of outliers, a predefined cutoff of 0.4 pg/mL was used, consistent with previously published data on p-tau217 as a biomarker for AD^14^. As individuals with DS differ from non-DS neurodevelopmental controls with respect to AD risk and amyloid/tau biology, univariable logistic regression analyses were also repeated as a DS-restricted sensitivity analysis including only participants with DSRD and DSwR, where cell counts permitted.

Figures were generated as jittered scatter plots with overlaid interquartile range (IQR; 25th–75th percentile) boxes and median lines. For visualization only, outliers were removed within relevant strata using the Tukey rule (values < Q1 − 1.5×IQR or >Q3 + 1.5×IQR), and y-axes were fixed to prespecified ranges to standardize presentation.

## Results

In total, 329 individuals were screened for participation in this study. Ultimately, 132 individuals were enrolled with 197 (59.9%) excluded due to meeting at least one exclusion criteria. Of those excluded, 165 (83.6%) had a monogenetic NDD, 16 (8.1%) elected to not participate in research, 14 (7.1%) were excluded because of incomplete data and 2 (1.0%) did not complete the necessary enrollment forms. Across 132 participants with available p-tau217 measurements, 48 (36.3%) were classified as DSRD, 39 (29.5%) as DSwR, and 45 (34.1%) as NDD Control. Baseline demographic and clinical characteristics by group are summarized in Table 1 and Supplementary Table 2. Participants with DSRD were older at collection (mean [SD], 19.4 [3.4] years) than those with DSwR (15.3 [5.8] years; mean difference, 4.10 years [95% CI, 2.19–6.29]; *p* < 0.001) and NDD Control (16.1 [4.8] years; mean difference, 3.20 years [95% CI, 1.46–4.94]; *p* < 0.001; overall *p* < 0.001). BMI was higher in DSRD (27.8 [7.1]) than in DSwR (23.9 [6.1]; mean difference, 3.90 [95% CI, 1.12–6.58]; *p* = 0.01) and NDD Control (23.7 [6.7]; mean difference, 4.10 [95% CI, 1.20–6.82]; *p* = 0.01; overall *p* = 0.01). Cardiac history was more common in DSRD (37.5%) and DSwR (35.9%) than in NDD Control (13.3%; risk difference vs NDD Control, 24.2 percentage points [95% CI, 7.3–41.1] and 22.6 percentage points [95% CI, 4.5–40.6], respectively; overall *p* = 0.01), and thyroid disease was similarly more frequent in DSRD (39.6%) and DSwR (38.5%) than in NDD Control (8.9%; risk difference vs NDD Control, 30.7 percentage points [95% CI, 14.6–46.8] and 29.6 percentage points [95% CI, 12.2–47.0]; overall *p* = 0.01). Obstructive sleep apnea (OSA), both untreated and treated, was more frequent in DSRD (52.1%) and DSwR (43.6%) than in NDD Control (15.6%; risk difference vs NDD Control, 36.5 percentage points [95% CI, 18.9–54.2] and 28.0 percentage points [95% CI, 9.2–46.8]; overall *p* = 0.01). Degree of ID distribution also differed across groups (overall *p* < 0.01), with no/minimal ID observed in 46.7% of NDD Control vs 0% in DSRD (risk difference, 46.7 percentage points [95% CI, 25.7–60.9]). A sub-analysis comparing ID severity between participants with DSRD and DSwR revealed no significant difference between the two cohorts (*p* = 0.19).

**Table 1:** Clinical and demographic data

Variable	DSRD (*n* = 48)	DSwR (*n* = 39)	NDD (*n* = 45)	All (*n* = 132)	*p* value
Male sex	22 (45.8%)	29 (74.4%)	23 (51.1%)	74 (56.1%)	0.12
Age (mean, SD)	19.4 (3.4)	15.3 (5.8)	16.1 (4.8)	17.1 (5.0)	<0.001
BMI (mean, SD)	27.8 (7.1)	23.9 (6.1)	23.7 (6.7)	25.3 (6.9)	0.01
Race					0.75
*White/Caucasian*	40 (83.3%)	32 (82.1%)	43 (95.6%)	115 (87.1%)	
*Black/African American*	4 (8.3%)	2 (5.1%)	2 (4.4%)	8 (6.1%)	
*Asian*	4 (8.3%)	5 (12.8%)	0 (0%	9 (6.8%)	
Hispanic ethnicity	15 (31.3%)	18 (46.2%)	16 (35.6%)	49 (37.1%)	0.59
Autism	10 (20.8%)	16 (41.0%)	20 (44.4%)	46 (34.8%)	<0.001
APOE status	9 (18.8%)	8 (20.5%)	5 (11.1%)	22 (16.7%)	0.81
*ε2/4*	1/9 (11.1%)	2/8 (25.0%)	0/5 (0%)	3/22 (13.6%)	
*ε3/4*	7/9 (63.6%)	5/8 (62.5%)	4/5 (80.0%)	16/22 (72.7%)	
*ε4/4*	1/9 (11.1%	1/8 (12.5%)	1/5 (20.0%)	3/22 (13.6%	
p-tau 217 (pg/mL) (median, IQR)	0.082 (0.063–0.101)	0.084 (0.069–0.129)	0.080 (0.063–0.115)	0.082 (0.064–0.112)	0.57
*DSRD clinical features*					
Age at onset (mean, SD)	18.7 (3.7)				
Clinical phenotype					
*AMS/Behavioral*	41 (85.4%)				
*Cognitive decline*	42 (87.5%)				
*Dev regression*	48 (100%)				
*Focal deficits/seizure*	3 (6.3%)				
*Language deficits*	39 (81.3%)				
*Movement disorder*	48 (100%)				
*Psychiatric symptoms*	42 (87.5%)				
*DSRD neurodiagnostic abnormalities*^a^					
EEG abnormal	15 (31.3%)				
*Global slowing*	13 (86.7%)				
*Focal slowing*	1 (6.7%)				
MRI abnormal	14 (29.2%)				
T2/FLAIR > 2 mm	2 (14.3%)				
GRE/SWI	13 (92.9%)				
Lumbar puncture abnormal	8 (16.7%)				
*Pleocytosis*	1 (12.5%)				
*Total protein elevated*	5 (62.5%)				
*IgG index elevated*	3 (37.5%)				
*Oligoclonal bands*	5 (62.5%)				

Continuous variables are mean (SD) unless otherwise specified; p-tau217 is median (IQR) due to right-skew. Between-cohort comparisons used one-way analysis of variance (ANOVA) for age and BMI, the Kruskal–Wallis test for p-tau217, and Fisher–Freeman–Halton exact tests for categorical variables. All tests were two-sided. The table presents unadjusted overall *p* values; Holm adjustment was applied only to post hoc pairwise comparisons of p-tau217, which are not presented in the table.

^a^Individuals could have more than one neurodiagnostic abnormality.

**Fig. 1:**
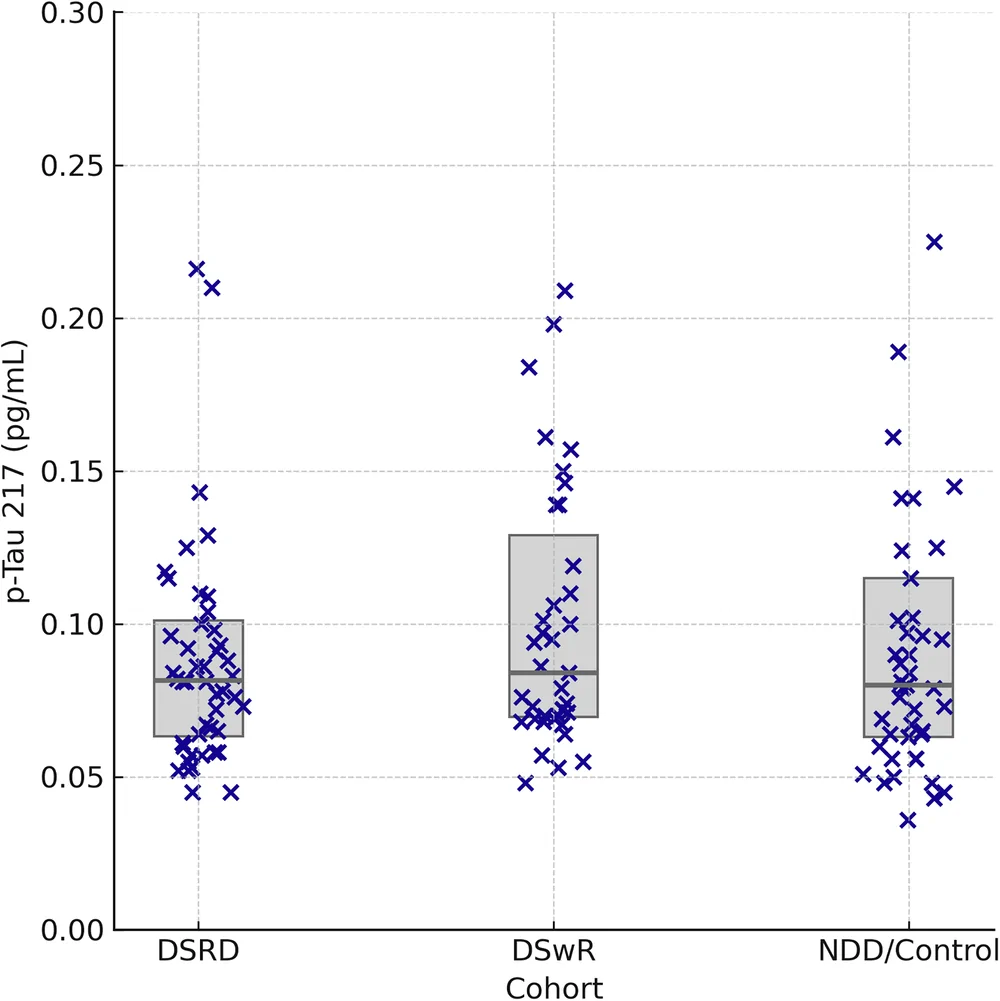
Plasma p-tau217 concentrations across clinical cohorts. Scatter plots with jitter display plasma p-tau217 values for individuals with Down syndrome regression disorder (DSRD), Down syndrome without regression (DSwR), and neurodevelopmental/control (NDD/Control) groups. Each point represents one biologically independent participant (one plasma sample per participant). Box plots are based on *n* = 48 DSRD, *n* = 39 DSwR, and *n* = 45 NDD/control biologically independent participants. For each cohort, a light gray box represents the interquartile range (IQR; 25th–75th percentile) with a darker gray border, and the horizontal line denotes the median p-tau217 value. Across cohorts, p-tau217 values largely overlapped with similar distributional characteristics and medians within a narrow range. For visualization, the y-axis was limited to 0.00–0.30 pg/mL; values outside this range are not shown, but all participants were included in calculating medians and IQRs and in all statistical analyses. Source data are provided in Supplementary Data 1.

*AMS* altered mental status, *BMI* body mass index, *CI* confidence interval, *DS* Down syndrome, *DSRD* Down syndrome regression disorder, *ID* intellectual disability, *IQR* interquartile range, *NDD* neurodevelopmental disorder, *OCD* obsessive compulsive disorder, *OSA* obstructive sleep apnea, *SD* standard deviation.

p-tau217 distributions were right-skewed but showed similar central tendencies across groups: DSRD median 0.082 pg/mL (IQR: 0.063–0.101), DSwR median 0.084 pg/mL (IQR: 0.069–0.129), and NDD Control median 0.080 pg/mL (IQR: 0.063–0.115) (Table 1 and Fig. 1). The proportion of APOE ε4 carriers was low and did not differ significantly between groups (*p* = 0.81). In the multivariable model, APOE carrier-negative vs carrier-positive status was not associated with plasma p-tau217 concentrations (11.3% difference; 95%: −26.3–67.5, *p* = 0.58) (Table 2).

**Table 2:** Adjusted percent difference in geometric mean p-tau217 associated with covariates

Predictor (vs reference)	% change in p-tau 217	95% CI	*p* value
No OSA v. Untreated OSA	27.1	−17.1–56.6	0.25
No psychiatric disease v. psychiatric disease	14.1	−24.0–61.9	0.49
No Epilepsy v. Epilepsy	12.7	−22.0–60.3	0.42
Non-White v. White Race	13.0	−18.5–56.6	0.46
APOE carrier negative v. APOE carrier positive	11.3	−26.3–67.5	0.58
No cardiac history v. cardiac history	12.4	−12.0–43.5	0.35
ASD v. No ASD	11.9	−12.8–43.7	0.38
DSwR v. NDD Control	11.0	−23.2–60.3	0.58
Female v. Male Sex	4.9	−15.1–29.6	0.66
BMI (per 1 kg/m^2^)	0.8	−1.7–3.5	0.53
Treated OSA v. Untreated OSA	−0.1	−33.6–50.5	1.00
No cancer history v. cancer history	−0.5	−68.8–227.7	0.99
No immunologic condition v. immunologic condition	−0.5	−33.4–48.7	0.98
DSwR v. DSRD	−1.7	−25.8–30.3	0.91
Age at collection (per 1 year)	−2.6	−6.2–1.1	0.17
No diabetes v diabetes	−6.4	−37.6–40.4	0.75
NDD v. DSRD	−11.4	−40.1–31.1	0.54
No thyroid disease v. thyroid disease	−17.5	−40.5–14.5	0.25
Mild ID v. No/Minimal ID	−23.8	−65.2–67.1	0.50
Moderate ID v no/minimal ID	−24.0	−64.3–61.9	0.48
Severe ID v. no/minimal ID	−28.9	−69.6–66.2	0.43

Estimates were derived from multivariable linear regression with log-transformed p-tau217 as the dependent variable and heteroskedasticity-consistent (HC3) robust standard errors. Regression coefficients were back-transformed and are presented as percentage differences in geometric mean p-tau217, calculated as 100 × [exp(β) − 1], with 95% confidence intervals derived from the robust standard errors. All tests were two-sided. Reported *p* values were not adjusted for multiple comparisons; estimates from the broader clinical model were exploratory.

*ASD* Autism spectrum disorder, *BMI* body mass index, *DS* Down syndrome, *DS-ASD* Down syndrome with autism spectrum disorder, *DSwR* Down syndrome without regression, *DSRD* Down syndrome regression disorder, *ID* intellectual disability, *NDD* neurodevelopmental disorder, *OSA* obstructive sleep apnea.

In unadjusted group comparisons, p-tau217 did not differ statistically by diagnostic group (Kruskal–Wallis *p* = 0.57; one-way ANOVA *p* = 0.51; Welch ANOVA *p* = 0.66). Pairwise comparisons likewise showed no evidence of group separation after multiplicity correction (Holm-adjusted *p* values: DSRD vs DSwR, *p* = 0.99; DSRD vs NDD Control, *p* = 0.99; DSwR vs NDD Control, *p* = 0.99). These findings are represented in Fig. 1.

In multivariable regression models estimating adjusted diagnostic group effects on log-transformed p-tau217 with robust standard errors, diagnostic group remained non-significant. In adjusted log-linear models geometric mean plasma p-tau217 did not differ for DSwR compared with DSRD (−1.7%; 95% CI: −25.8–30.3; *p* = 0.91) or for NDD controls compared with DSRD (−11.4%; 95% CI: −40.1–31.1; *p* = 0.54). Likewise, p-tau217 did not differ between DSwR and NDD controls (+11.0%; 95% CI: −23.2–60.3; *p* = 0.58).

**Fig. 2:**
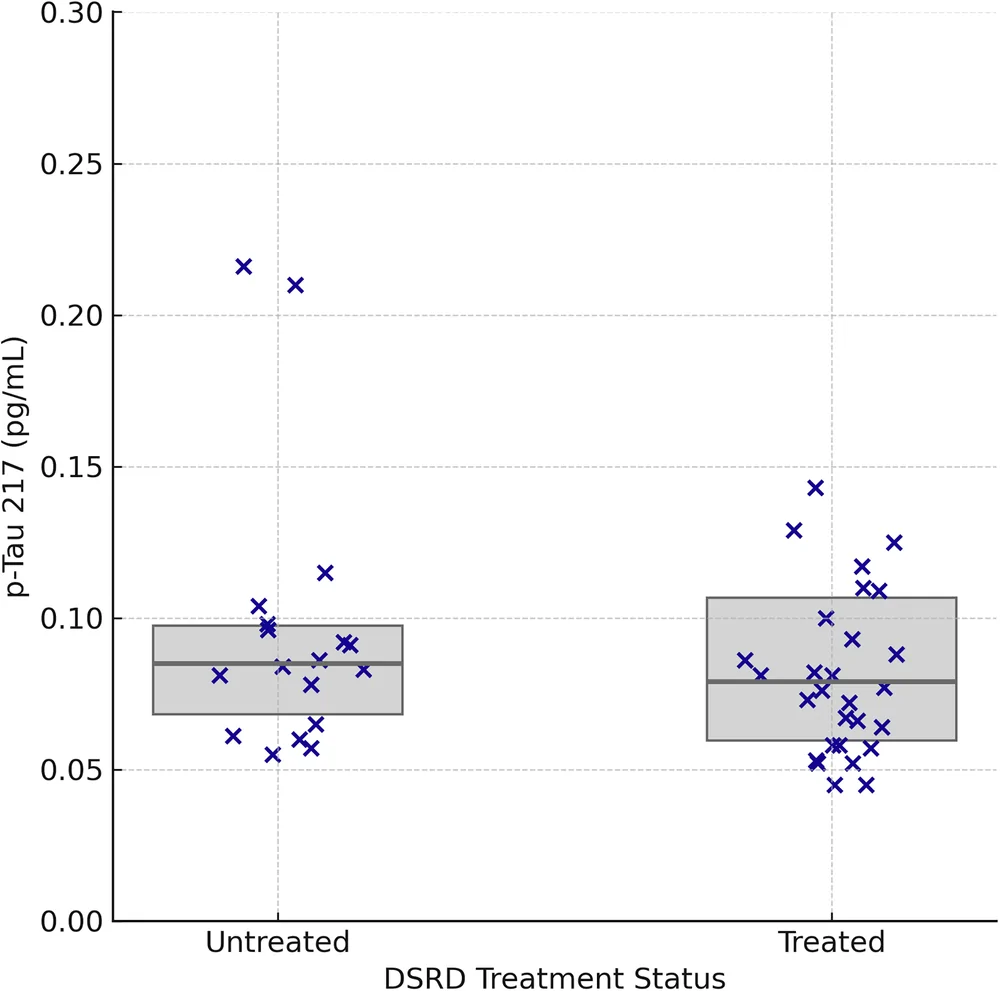
Plasma p-tau217 in treated and untreated DSRD cohorts. Scatter plots with jitter illustrate p-tau217 concentrations among individuals with Down syndrome regression disorder stratified by treatment status. Treatment was defined as receipt of psychotropic medication and/or immunotherapy. Each point represents one biologically independent participant (one plasma sample per participant). Box plots are based on *n* = 18 untreated and *n* = 30 treated biologically independent participants with DSRD. Light gray rectangles denote the interquartile range (25th–75th percentile) for each group, outlined in darker gray, with the median indicated by a horizontal line. Treated and untreated groups show overlapping p-tau217 distributions with comparable medians. For visualization, the y-axis was limited to 0.00–0.30 pg/mL; values outside this range are not shown, but all participants were included in calculating medians and IQRs and in all statistical analyses. Source data are provided in Supplementary Data 2.

**Fig. 3:**
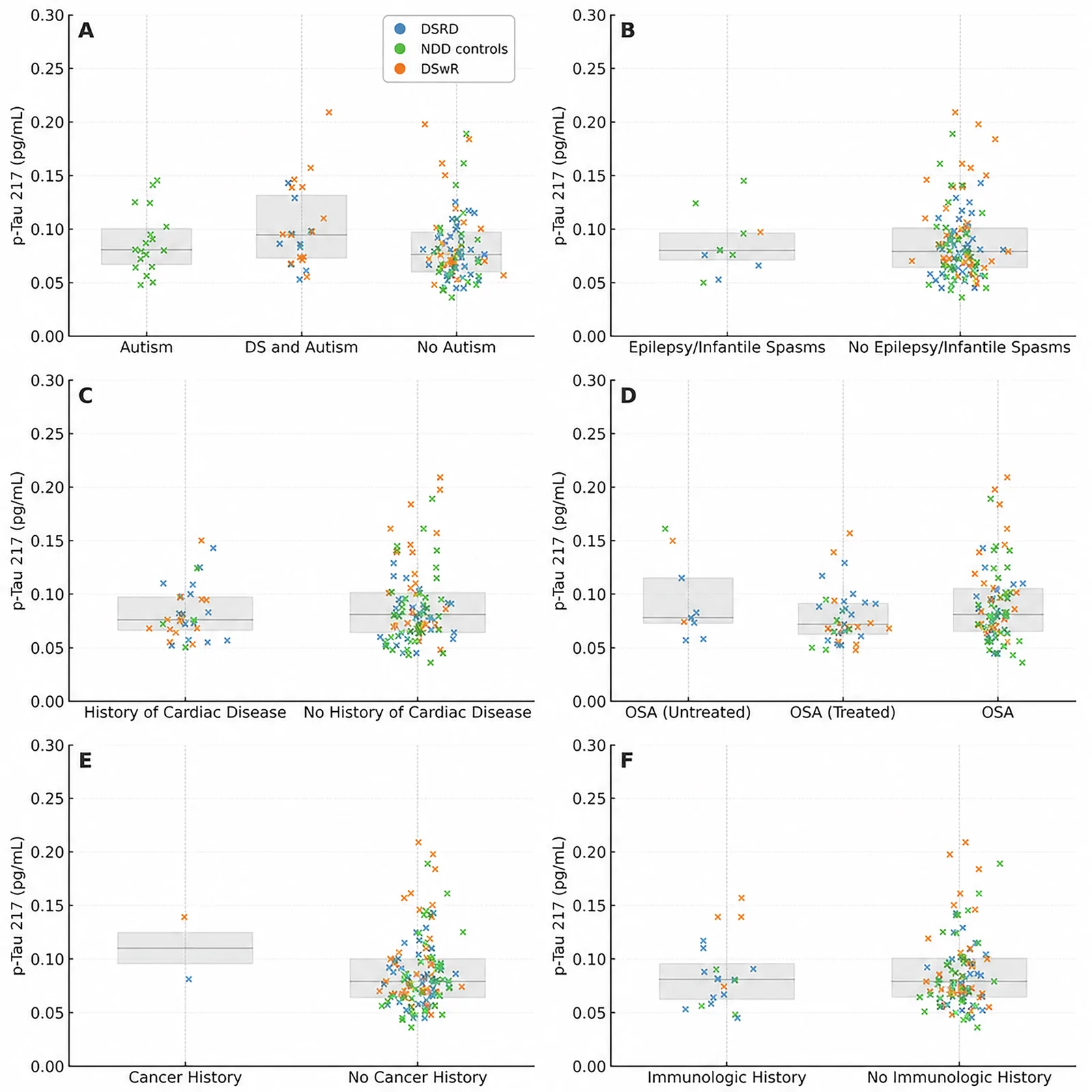
Plasma p-tau217 concentrations across clinical and demographic characteristics. Scatterplots (jittered) show individual participants’ plasma p-tau217 concentrations (pg/mL) across key clinical strata. Blue crosses indicate DSRD, orange crosses indicate DSwR, and green crosses indicate NDD controls. Each point represents one biologically independent participant (one plasma sample per participant). Box plots are based on the following numbers of biologically independent participants: **A** *n* = 20 with autism, *n* = 25 with Down syndrome and autism, and *n* = 87 without autism; **B** *n* = 11 with epilepsy or infantile spasms and *n* = 121 without epilepsy or infantile spasms; **C** *n* = 34 with a cardiac history and *n* = 98 without a cardiac history; **D** *n* = 9 with untreated obstructive sleep apnea (OSA), *n* = 36 with treated OSA, and *n* = 87 without OSA; **E** *n* = 2 with a cancer history and *n* = 130 without a cancer history; and **F** *n* = 22 with an immunologic disorder history and *n* = 110 without an immunologic disorder history. Overlaid boxes indicate the interquartile range (25th–75th percentiles) with the median shown as the central horizontal line. For visualization, the *y*-axis was limited to 0.00–0.30 pg/mL; values outside this range are not shown, but all participants were included in calculating medians and IQRs and in all statistical analyses. Source data are provided in Supplementary Data 3.

Among participants with DSRD, p-tau217 levels were similar when stratified by treatment status (Fig. 2), with no visually apparent separation between treated and untreated categories. There was similarly no effect observed on p-tau217 levels for individuals with DSRD and EEG abnormalities (*p* = 0.67), neuroimaging abnormalities (*p* = 0.11) and abnormal CSF (*p* = 0.36). In exploratory univariable screening of candidate predictors, which was corrected for multiple testing using Benjamini–Hochberg false discovery rate control, none demonstrated statistically significant associations with p-tau217; the smallest nominal associations included age at collection (Spearman *ρ* = −0.139; *p* = 0.11), obstructive sleep apnea (*p* = 0.07), and autism status (*p* = 0.07). Correspondingly, Fig. 3A–F shows overlapping p-tau217 distributions across autism, epilepsy/infantile spasms, cardiac history, sleep apnea, cancer history, and immunologic disorder categories, without consistent divergence by diagnostic group. In the broader exploratory clinical model, no individual covariate was significantly associated with plasma p-tau217, although these estimates were interpreted cautiously given the number of parameters relative to cohort size (Table 2).

**Fig. 4:**
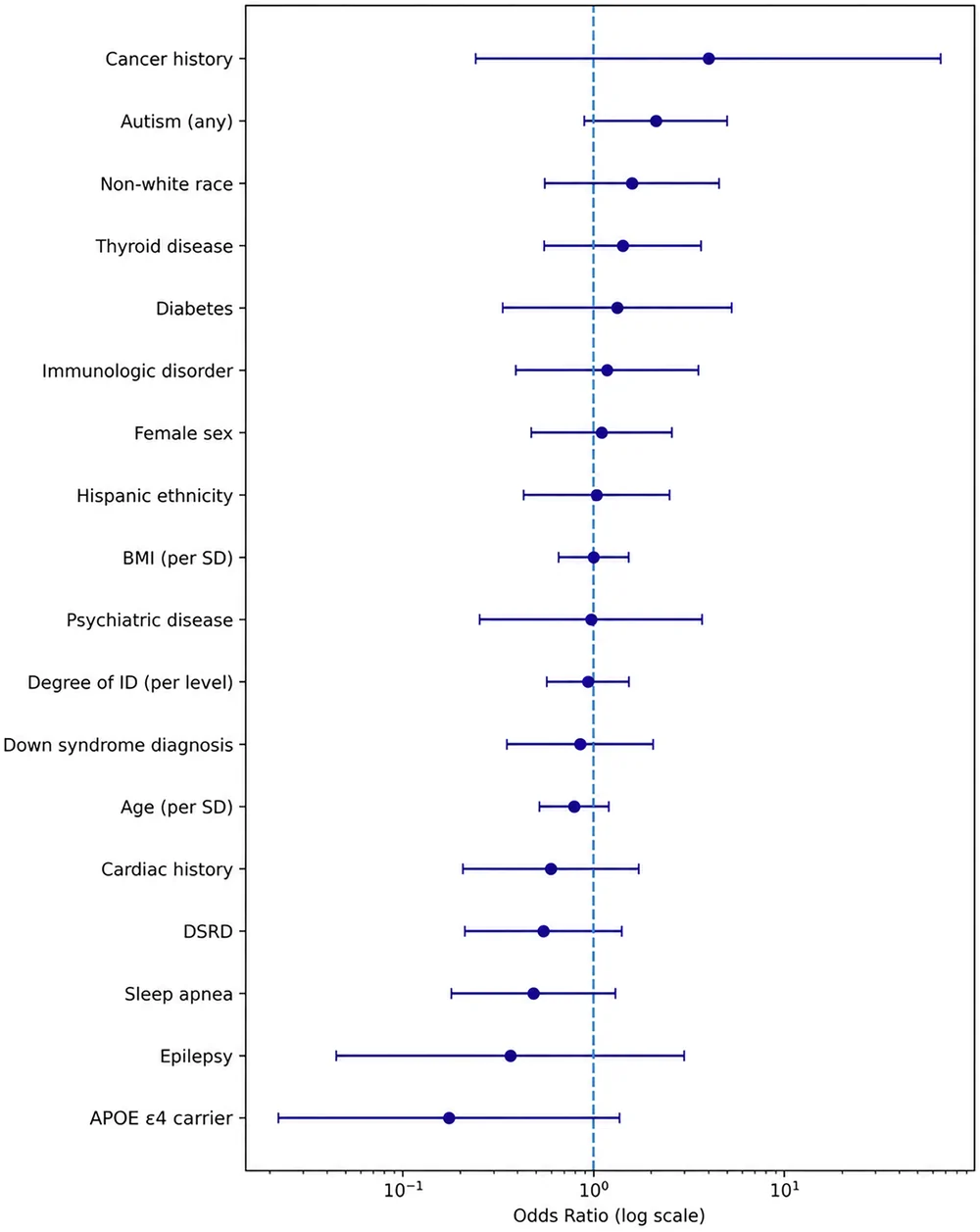
Odds ratios for elevated p-Tau217 across clinical and demographic variables. Forest plot displaying univariable odds ratios (ORs) and 95% Wald confidence intervals (CIs) for associations between demographic or clinical characteristics and elevated plasma p-tau217, defined as ≥0.125 pg/mL. Each univariable logistic regression model included *n* = 132 biologically independent participants. The filled circle at the center of each horizontal error bar represents the point estimate of the OR from the corresponding univariable logistic regression model; it does not represent a mean or median. Horizontal error bars represent 95% Wald CIs. ORs are displayed on a logarithmic scale, and the dashed vertical line indicates the null value (OR = 1). Age and body mass index (BMI) were modeled per 1-standard-deviation increase, and degree of intellectual disability (ID) was modeled per level. None of the evaluated variables was significantly associated with elevated p-tau217. Sleep apnea showed a nonsignificant trend toward lower odds, whereas autism showed a nonsignificant trend toward higher odds. Source data, including the odds ratios and corresponding 95% confidence intervals, are provided in Supplementary Data 4.

In exploratory univariable logistic models evaluating elevated p-tau217 levels, no clinical or demographic variable demonstrated a statistically significant association. These analyses were hypothesis-generating, and nominal associations were interpreted cautiously given the number of comparisons. No association remained statistically significant after Benjamini–Hochberg false discovery rate correction. Findings were similar in a DS-restricted sensitivity analysis including only participants with DSRD and DSwR, with no clinical or demographic variable demonstrating a statistically significant association with elevated p-tau217. OSA showed a nonsignificant trend toward lower odds of elevated p-tau217 (OR 0.48; 95% CI 0.18–1.30; *p* = 0.15), whereas autism demonstrated a nonsignificant trend toward higher odds (OR 2.12; 95% CI 0.89–5.01; *p* = 0.09). APOE carrier status demonstrated a stronger but still nonsignificant inverse association (OR 0.17; 95% CI 0.02–1.36; *p* = 0.10). All other variables, including primary diagnosis, psychiatric disease, cardiac history, diabetes, thyroid disease, and degree of intellectual disability (ID), were not associated with elevated p-tau217 (Fig. 4 and Table 3).

**Table 3:** Odds ratios for elevated p-tau217 (≥0.125 pg/mL)

Variable	Odds Ratio	95% CI	*p* value
Cancer history	4.00	0.24–66.11	0.33
Autism (any)	2.12	0.89–5.01	0.09
Non-white race	0.59	0.34–5.31	0.39
DSwR	1.54	0.63–3.76	0.34
Thyroid disease	1.42	0.55–3.65	0.47
Diabetes (any type)	1.33	0.34–5.31	0.68
Immunologic disorder	1.18	0.39–3.54	0.77
Female sex	1.10	0.47–2.57	0.82
Hispanic ethnicity	1.04	0.43–2.49	0.94
BMI (per SD)	1.00	0.66–1.53	1.00
Psychiatric disease	0.97	0.25–3.71	0.96
Degree of ID (per level)	0.94	0.57–1.54	0.79
Age (per SD)	0.79	0.52–1.20	0.27
Cardiac history	0.60	0.21–1.72	0.34
DSRD	0.55	0.21–1.41	0.21
OSA	0.48	0.18–1.30	0.15
Epilepsy	0.37	0.04–2.99	0.35
APOE carrier	0.17	0.02–1.36	0.10

Odds ratios were estimated using separate univariable logistic regression models, with elevated p-tau217 (>0.125 pg/mL) as the binary dependent variable. All tests were two-sided, and 95% Wald confidence intervals are presented. The *p* values shown are unadjusted. The Benjamini–Hochberg procedure was additionally applied across the exploratory models to control the false discovery rate; no association remained statistically significant after this adjustment.

*BMI* body mass index, *DSwR* Down syndrome without regression, *DSRD* Down syndrome regression disorder, *ID* intellectual disability, *OSA* obstructive sleep apnea.

Three high-value outliers with p-tau217 > 0.4 pg/mL were identified in this cohort. These included two participants (2/45, 4.4%) in the NDD cohort (1.454 pg/mL and 0.575 pg/mL) and one participant (1/48, 2.1%) in the DSRD cohort (0.686 pg/mL). Repeat p-tau217 measurements were performed for all outliers using the original sample and were 1.379 pg/mL (−5.2%), 0.611 pg/mL ( + 5.9%), and 0.634 pg/mL (−7.6%), respectively. There were no DSwR participants who exceeded this threshold. All three outliers were female. The two NDD control outliers were younger (ages 6 and 9 years) with low BMI (average 16.5 kg/m²), whereas the DSRD outlier was older (23 years) with severe obesity (BMI 52.6 kg/m²) and treated OSA. APOE positivity was present in one outlier (33%) and absent in the others. None had clinical phenotypes or neuroimaging consistent with AD.

## Discussion

This study provides evidence that DSRD is not associated with the biomarker signature of AD. Despite clinical similarities that can sometimes blur the distinction between DSRD and early-onset AD in individuals with DS, our findings demonstrate that plasma p-tau217, a sensitive and specific early biomarker for AD pathology, remains unremarkable in individuals with DSRD. Levels of p-tau217 in DSRD were indistinguishable from those in DSwR and NDD controls, even after adjusting for demographic and clinical variables.

These results challenge a clinical bias to attribute regression in DS to underlying AD. The absence of elevated p-tau217 in DSRD, despite the older age and greater medical comorbidity of this group, argues against an AD-like tauopathy as the primary driver of regression. This is further supported by previous neuroimaging and CSF biomarker studies, which also fail to show an AD-like profile in DSRD^10,16–18^. This is clinically meaningful as mislabeling DSRD as AD may delay or misdirect clinical evaluation and management, potentially overlooking treatable neuroimmune or neuropsychiatric causes. Consequently, clinicians should exercise caution when attributing rapid regression in young adults with DS to neurodegeneration. Our findings reinforce the need to conceptualize DSRD as a distinct clinical and biological entity, separate from AD, and to prioritize research into alternative mechanisms, particularly neuroimmune and neuropsychiatric pathways.

The lack of p-tau217 in individuals with DSRD at the group level does not definitively rule out DS-AD although when taken in the context of previously published neuroimaging^17,18^ and CSF biomarkers^16,17^, a growing amount of clinical and biomarker data now differentiates DSRD from DS-AD. These findings are markedly different than what is observed in individuals with DS-AD^19–23^ supporting the hypothesis that this study’s p-tau217 null finding is supportive of distinct pathophysiology rather than a failure of measurement. Importantly, the treatment of DSRD with psychotropics and/or immunotherapy was also not associated with differences in p-tau217 levels, making a treatment effect of the biomarker less likely. This study also explored the potential impact of medical comorbidities present in individuals with DSRD and DSwR. The stability of p-tau217 levels across diverse clinical strata further reinforces the specificity of our findings. Despite the DSRD cohort being significantly older and having a higher prevalence of medical comorbidities such as OSA and thyroid disease, these factors did not result in an AD-like biomarker signature. This suggests that the p-tau217 profile in DSRD is robust against common systemic and neurodevelopmental confounders found in the DS population.

The presence of three participants with elevated p-tau217 values warrants discussion. Outliers all had confirmation testing performed using an identical protocol, all with repeat values within +/-10% of the initial value. These individuals did not have any shared clinical phenotypes such as more severe intellectual impairment, medical comorbidities, or older age. Further, the elevated level in the DSRD patient was from an individual with mild-to-moderate disease. This same individual with DSRD was noted to have a typical MRI (neither global/hippocampal atrophy nor SWI signal abnormalities in the basal ganglia present) and typical CSF. Interestingly, the two highest values were in two young individuals from the NDD cohort, neither of whom had CSF available for testing. While no patient had clinical evidence of AD pathology and had minimal ID at baseline, tau species elevations have been reported in individuals with NDDs in the past^24,25^, although this literature is inconsistent^26^. The few high-value outliers identified lacked clinical or neuroimaging phenotypes consistent with AD. In these rare instances, p-tau217 elevation may represent acute neuronal insult or temporal metabolic factors rather than chronic tauopathy. For example, the DSRD outlier’s extreme BMI (52.6) and treated OSA highlight the need for cautious interpretation of peripheral biomarkers in medically complex individuals, even when group-level data remains overwhelmingly negative.

Placing these findings in the context of recently published DS-AD biomarker dynamics further supports this interpretation. Huber et al. demonstrated that plasma p-tau217 strongly discriminates cognitively stable individuals with DS from those with prodromal AD and AD dementia and also distinguishes amyloid-positive from amyloid-negative individuals with DS^13^. In that study, p-tau217 increased across the DS-AD clinical and biomarker continuum, supporting its role as a marker of AD-related amyloid/tau pathology in DS. By contrast, p-tau217 values in the present cohort were low and highly overlapping across DSRD, DSwR, and NDD controls, with median values of 0.082, 0.084, and 0.080 pg/mL, respectively. Although direct numerical comparison across studies should be made cautiously because p-tau217 concentrations vary by assay platform and analytic method, the distribution observed here does not resemble the biomarker pattern expected in symptomatic DS-AD. These findings therefore support the interpretation that DSRD, at least in this young cohort, is not characterized by the plasma p-tau217 dynamics typical of AD progression in DS.

This study is not without limitations. First, while the total recruitment was sizable for a clinically characterized DSRD cohort, the total number of individuals with DSRD and DSwR limits statistical precision for between-group contrasts and multivariable models, as reflected in wide confidence intervals for adjusted group effects. Modest true differences in p-tau217 across groups, or effects restricted to specific subgroups, may therefore have been missed. In addition, a “positive control” population of individuals with DS-AD was not available for comparison, although published data on p-tau217 in individuals with DS-AD have been reported^22,23,27^. Importantly, individuals with amyloid negative-asymptomatic DS, amyloid positive-asymptomatic DS, prodromal DS-AD, and DS-AD had median values of 0.4, 0.9, 1.8, and 2.5 pg/mL^27^, respectively. Compared to our cohort, p-tau217 values were over 22 times greater in the prodromal DS-AD group and 31 times greater in the DS-AD cohorts, reflecting marked differences between DS-AD and our cohorts. Secondly, this dataset was also derived from a single site, which increases the risk of selection bias and severity bias across all cohorts. Third, biomarker assessment was cross-sectional, with a single p-tau217 measurement per participant. Timing in relation to symptom onset, peak severity, recovery, and treatment initiation was not uniformly captured in the analytic dataset. Consequently, the analysis cannot determine whether transient biomarker changes occur during acute regression or whether biomarker trajectories change with treatment response. Future studies including longitudinal assessment of p-Tau217 would be highly valuable. Fourth, p-tau217 represents one component of the AD biomarker cascade and does not comprehensively exclude AD biology. The absence of a group-level p-tau217 signal does not rule out amyloid positivity, coexisting neurodegeneration, or mixed pathologies in individual participants, nor does it substitute for multimodal profiling with complementary blood biomarkers (eg, Aβ42/40, p-tau181, GFAP, NfL), cerebrospinal fluid measures, or amyloid/tau PET imaging when clinically indicated. Future studies will need to include more in-depth analysis of these other biomarkers. Similarly, there are no established p-tau217 cutoffs specific to individuals with DS^13^ and, as such, a more stringent 0.125 pg/mL was used^15^, increasing the risk of false positive results in individuals with DS and DSwR. As such, these data should be interpreted as hypothesis-generating. Finally, the study design is observational, and residual confounding (including medication exposures, baseline cognitive level, psychiatric comorbidity severity, and assay pre-analytic factors) cannot be excluded. Although the primary adjusted model was intentionally parsimonious, the broader exploratory clinical model included numerous comorbidities to evaluate whether the diagnostic group effect was robust to medical complexity. Because the broader clinical model included numerous covariates relative to the cohort size, it was at risk of overfitting and unstable estimates. These exploratory results should therefore be interpreted as hypothesis-generating rather than confirmatory. Taken together, these limitations highlight the need for larger, age-stratified, longitudinal studies with standardized phenotyping and multimarker biomarker panels to more definitively delineate whether and when neurodegenerative biology overlaps with DSRD phenotypes.

In summary, plasma p-tau217 did not differ meaningfully among individuals with DSRD, DSwR, and NDD, with concordant adjusted and sensitivity analyses. These findings argue against a prominent Alzheimer-type tau signal as a group-level driver of DSRD, while underscoring the limits of a single peripheral biomarker in a heterogeneous syndrome. Longitudinal studies integrating serial plasma measures with CSF, neuroimaging, treatment exposure, and complementary markers of neuroinflammation, synaptic injury, and blood–brain barrier dysfunction are needed to define biologically coherent DSRD subtypes and guide mechanism-based therapy.

## Supplementary information

Supplementary Materials

Description of Additional Supplementary files

Supplementary Data 1

Supplementary Data 2

Supplementary Data 3

Supplementary Data 4

## Data Availability

Deidentified source data underlying Fig. 1 are provided in Supplementary Data 1; source data underlying Fig. 2 are provided in Supplementary Data 2; source data underlying Fig. 3 are provided in Supplementary Data 3; and source data underlying Fig. 4 are provided in Supplementary Data 4. Additional participant-level data is not publicly available because the study population includes individuals with rare neurodevelopmental conditions and the combination of detailed demographic, clinical, genetic, and neurodiagnostic information could permit participant re-identification. Qualified investigators may request access by contacting the corresponding author (santoroj@usc.edu). Requests will be reviewed by the study investigators and the Children’s Hospital Los Angeles Institutional Review Board within 30 days. Approved access will require execution of a data use agreement limiting use to the approved research purposes.

## Code availability

No custom code or mathematical algorithms were used in this study. All analyses were conducted using established methods and standard software as indicated in the “Methods” section.
