# Supplementary material for: Phosphorylated tau 217 in young adults with Down syndrome and Down syndrome regression disorder
Source: Commun Med (Lond). 2026 Sep 21;6:541. doi: 10.1038/s43856-026-01921-y (PMC13680608; doi:10.1038/s43856-026-01921-y)
Supplement: Supplementary file 1 [file 43856_2026_1921_MOESM1_ESM.pdf]

Supplementary Table 1: Determination of Abnormalities on Neurodiagnostic Studies

**Neurodiagnostic Test**	**Definition of Abnormal**
Electroencephalogram (EEG)	1. Focal or generalized slowing, focal epileptiform discharges out of any cortex, or seizure were considered abnormal.<br>2. Generalized discharges were considered abnormal although inconsistent with the diagnosis of DSRD.<br>3. All individuals had to have at least one prior EEG prior to the onset of DSRD symptoms that did not demonstrate these results.
Magnetic Resonance Imaging (MRI)	1. T1: Abnormalities on T1 sequencing were defined as structural abnormalities of gray matter, white matter, brainstem, or cerebellum. Definition of hypoplasia of a structure was a region demonstrating volume loss but not structural defects and was assessed subjectively (quantitative volumetry not performed).<br>2. T2: Abnormalities on T2/FLAIR sequencing were defined as lesions of gray matter or white matter measuring greater than 2 mm in diameter. Imaging abnormalities had to be present on both T2 and FLAIR images and had to be present on a two different field view (axial, sagittal, or coronal) to be defined as abnormal.<br>3. SWI: Abnormalities on SWI sequences were defined as any signal abnormality of any size or distribution in the gray or white matter structures. Anatomical variants in the cerebrovasculature detected on SWI (e.g., hypoplastic vessel) were not considered abnormal unless they were deemed to be potentially clinically significant (e.g., narrowing of the internal carotid arteries consistent with moyamoya disease).
Lumbar Puncture (LP)	1. Abnormalities were defined as having any of the following findings on a cerebrospinal fluid analysis:<br>a. White blood cell (WBC) count > 5 cells/mm3<br>b. Total protein > 60 mg/dL<br>c. Presence of oligoclonal bands<br>d. An immunoglobulin G (IgG) index of > 0.66,<br>e. Neopterin elevation (> 33 nmol/mL).<br>i. Samples with over 1,000 red blood cells (RBC) were excluded from analysis.

Legend: Down Syndrome Regression Disorder (DSRD); electroencephalogram (EEG)

Supplementary Table 2: Additional clinical and demographic data

**Variable**	**DSRD** (n = 48)	**DSwR** (n = 39)	**NDD** (n = 45)	**All** (n = 132)	***p* Value**
Epilepsy/Infantile Spasms	4 (8.3%)	2 (5.1%)	7 (15.6%)	13 (9.8%)	0.19
Cardiac History	18 (37.5%)	14 (35.9%)	6 (13.3%)	38 (28.8%)	0.01
OSA	25 (52.1%)	17 (43.6%)	7 (15.6%)	49 (37.1%)	0.01
Untreated	7/25 (28.0%)	5/17 (29.4%)	1/7 (14.3%)	13/49 (26.5%)	
Treated	18/25 (72.0%)	12/17 (70.6%)	6/7 (85.7%)	36/49 (73.4%)	
Cancer History	1 (2.1%)	1 (2.6%)	0 (0%)	2 (1.5%)	0.61
Immunologic History $^{\alpha}$	12 (25.0%)	5 (12.8%)	6 (13.3%)	23 (17.4%)	0.18
Thyroid Disease	19 (39.6%)	15 (38.5%)	4 (8.9%)	38 (28.8%)	0.01
Degree of ID $^{\beta}$					<0.001
None/Minimal	0 (0%)	0 (0%)	21 (46.7%)	21 (15.9%)	
Mild	22 (45.8%)	16 (41.0%)	12 (26.7%)	50 (37.9%)	
Moderate	24 (50.0%)	17 (43.6%)	10 (22.2%)	56 (42.4%)	
Severe	2 (4.2%)	6 (15.4%)	2 (4.4%)	5 (3.8%)	
Hypertension	0 (0%)	0 (0%)	1 (2.2%)	1 (0.7%)	0.35
Diabetes	7 (14.5%)	4 (10.3%)	1 (2.2%)	12 (9.1%)	0.12
Type I Diabetes	5/7 (71.4%)	2/4 (50.0%)	0/1 (0%)	7/12 (58.3%)	
Type II Diabetes	2/7 (28.6%)	2/4 (50.0%)	1/1 (100%)	5/12 (41.7%)	
Prior Psychiatric Disease $^{\gamma}$	3 (6.3%)	4 (10.3%)	9 (20%)	16 (12.1%)	0.09
Anxiety	1/3 (33.3%)	1/4 (25.0%)	5/9 (55.5%)	7/16 (43.8%)	
Depression	1/3 (33.3%)	1/4 (25.0%)	2/9 (22.2%)	4/16 (25.0%)	
OCD	1/3 (33.3%)	2/4 (50.0%)	2/9 (22.2%)	5/16 (31.3%)	
DSRD Clinical Features					
Probable DSRD					
Possible	18 (37.5%)				
Probable	30 (62.5%)				
Days to Symptom Nadir (mean, SD)	38.3 (15.9)				

Legend: Down Syndrome Regression Disorder (DSRD), Down syndrome without regression (DSwR), Neurodevelopmental disorder (NDD), Standard deviation (SD), α: excludes DSRD as an immunologic disorder, β: degree of intellectual disability reflects baseline (pre-regression) functioning in DSRD participants, γ: excludes DSRD as a psychiatric disorder.
