# Supplementary material for: Phosphorylated tau 217 in young adults with Down syndrome and Down syndrome regression disorder
Source: Commun Med (Lond). 2026 Sep 21;6:541. doi: 10.1038/s43856-026-01921-y (PMC13680608; doi:10.1038/s43856-026-01921-y)
Supplement: Supplementary file 2 [file 43856_2026_1921_MOESM2_ESM.docx]

Description of Additional Supplementary Files

File name: Supplementary Data 1-4

Description: Source data
